# Supplementary material for: Geospatial epidemiology of hospitalized patients with a positive influenza assay: A nationwide study in Iran, 2016–2018
Source: PLoS One. 2022 Dec 13;17(12):e0278900. doi: 10.1371/journal.pone.0278900 (PMC9747007; doi:10.1371/journal.pone.0278900)
Supplement: S1 Appendix — (DOCX) [file pone.0278900.s001.docx]

| ID | Province Name | Total PCR Test (n) | Positive PCR Result  (Total Case) (n) | The ratio of positive PCR tests to total PCR tests (%) | Cumulative Incidence * (CI) | Mortality (n) | CFR (%) | Female (n) | Male (n) | CVD (n) | Diabetes (n) | CRD (n) | CLD (n) | COPD (n) | Malignancy (n) | Obesity II (n) | Pregnancy (n) |
| --- | --- | --- | --- | --- | --- | --- | --- | --- | --- | --- | --- | --- | --- | --- | --- | --- | --- |
| 1 | **Alborz** | 3097 | 228 | 7.36 | 8.66 (7.38-9.94) | 14 | 6.14 | 155 | 73 | 24 | 12 | 5 | 1 | 21 | 0 | 1 | 58 |
| 2 | **Ardabil** | 646 | 90 | 13.93 | 7.16 (5.76-8.56) | 2 | 2.22 | 58 | 32 | 5 | 5 | 0 | 0 | 6 | 0 | 0 | 13 |
| 3 | **Azerbaijan East** | 3424 | 450 | 13.14 | 11.77 (10.49-13.05) | 41 | 9.11 | 232 | 218 | 68 | 31 | 14 | 1 | 65 | 7 | 7 | 27 |
| 4 | **Azerbaijan West** | 3287 | 306 | 9.31 | 9.37 (8.38-10.36) | 22 | 7.19 | 172 | 134 | 40 | 20 | 5 | 1 | 42 | 2 | 1 | 26 |
| 5 | **Bushehr** | 694 | 31 | 4.47 | 2.66 (1.87-3.45) | 3 | 9.68 | 16 | 15 | 3 | 1 | 1 | 0 | 3 | 0 | 0 | 1 |
| 6 | **Chaharmahal & Bakhtiari** | 1961 | 331 | 16.88 | 34.92 (31.36-38.48) | 9 | 2.72 | 161 | 170 | 36 | 22 | 12 | 2 | 43 | 3 | 4 | 14 |
| 7 | **Fars** | 2840 | 137 | 4.82 | 2.89 (2.39-3.39) | 8 | 5.84 | 60 | 77 | 28 | 13 | 8 | 4 | 17 | 0 | 3 | 4 |
| 8 | **Gilan** | 4237 | 316 | 7.46 | 12.64 (11.18-14.10) | 15 | 4.75 | 182 | 134 | 51 | 44 | 8 | 1 | 43 | 3 | 11 | 28 |
| 9 | **Golestan** | 1394 | 165 | 11.84 | 8.83 (7.58-10.08) | 3 | 1.82 | 105 | 60 | 18 | 9 | 2 | 2 | 18 | 1 | 5 | 46 |
| 10 | **Hamadan** | 3052 | 623 | 20.41 | 36.07 (33.13-39.01) | 25 | 4.01 | 334 | 289 | 89 | 39 | 21 | 6 | 94 | 6 | 6 | 35 |
| 11 | **Hormozgan** | 1773 | 41 | 2.31 | 2.31 (1.7-2.92) | 2 | 4.88 | 26 | 15 | 6 | 2 | 0 | 0 | 3 | 0 | 0 | 4 |
| 12 | **Ilam** | 307 | 55 | 17.92 | 9.48 (7.28-11.68) | 2 | 3.64 | 28 | 27 | 5 | 1 | 1 | 0 | 2 | 0 | 2 | 1 |
| 13 | **Isfahan** | 8999 | 434 | 4.82 | 8.49 (7.71-9.27) | 28 | 6.45 | 248 | 186 | 65 | 54 | 18 | 10 | 59 | 12 | 11 | 85 |
| 14 | **Kerman** | 8627 | 513 | 5.95 | 16.59 (14.87-18.31) | 29 | 5.65 | 307 | 206 | 58 | 35 | 13 | 3 | 86 | 8 | 2 | 69 |
| 15 | **Kermanshah** | 1010 | 147 | 14.55 | 7.68 (6.41-8.95) | 6 | 4.08 | 92 | 55 | 12 | 5 | 4 | 1 | 16 | 4 | 2 | 20 |
| 16 | **Khorasan North** | 916 | 240 | 26.20 | 28.15 (24.50-31.80) | 3 | 1.25 | 123 | 117 | 20 | 12 | 9 | 1 | 23 | 0 | 0 | 20 |
| 17 | **Khorasan Razavi** | 2725 | 787 | 28.88 | 12.31 (11.41-13.21) | 13 | 1.65 | 433 | 354 | 74 | 49 | 20 | 12 | 101 | 4 | 10 | 65 |
| 18 | **Khorasan South** | 305 | 69 | 22.62 | 8.97 (7.09-10.85) | 5 | 7.25 | 47 | 22 | 14 | 1 | 0 | 0 | 7 | 0 | 1 | 6 |
| 19 | **Khuzestan** | 2473 | 214 | 8.65 | 4.56 (3.97-5.15) | 16 | 7.48 | 110 | 104 | 36 | 18 | 7 | 1 | 26 | 1 | 3 | 26 |
| 20 | **Kohgiluyeh & Boyer Ahmad** | 641 | 12 | 1.87 | 1.68 (0.96-2.40) | 1 | 8.33 | 4 | 8 | 1 | 0 | 1 | 0 | 1 | 0 | 0 | 1 |
| 21 | **Kurdistan** | 1515 | 292 | 19.27 | 18.4 (16.24-20.56) | 14 | 4.79 | 179 | 113 | 34 | 15 | 6 | 1 | 29 | 2 | 4 | 48 |
| 22 | **Lorestan** | 653 | 85 | 13.02 | 4.88 (3.9-5.86) | 3 | 3.53 | 57 | 28 | 17 | 3 | 4 | 1 | 7 | 2 | 1 | 12 |
| 23 | **Markazi** | 1204 | 137 | 11.38 | 9.58 (8.11-11.05) | 2 | 1.46 | 83 | 54 | 16 | 6 | 4 | 0 | 19 | 4 | 4 | 26 |
| 24 | **Mazandaran** | 1712 | 273 | 15.95 | 8.47 (7.38-9.56) | 13 | 4.76 | 173 | 100 | 37 | 34 | 7 | 1 | 39 | 6 | 2 | 57 |
| 25 | **Qazvin** | 3123 | 847 | 27.12 | 66.57 (62.17-70.97) | 19 | 2.24 | 434 | 413 | 155 | 88 | 32 | 13 | 107 | 13 | 17 | 41 |
| 26 | **Qom** | 908 | 71 | 7.82 | 5.73 (4.35-7.11) | 5 | 7.04 | 41 | 30 | 10 | 4 | 3 | 0 | 10 | 1 | 0 | 15 |
| 27 | **Semnan** | 630 | 72 | 11.43 | 10.25 (8.14-12.36) | 1 | 1.39 | 44 | 28 | 12 | 4 | 3 | 0 | 7 | 0 | 0 | 13 |
| 28 | **Sistan & Baluchestan** | 844 | 199 | 23.58 | 7.24 (6.24-8.24) | 10 | 5.03 | 100 | 99 | 15 | 17 | 8 | 2 | 20 | 2 | 1 | 6 |
| 29 | **Tehran** | 16318 | 1667 | 10.22 | 12.71 (11.98-13.44) | 75 | 4.5 | 875 | 792 | 336 | 210 | 89 | 21 | 255 | 47 | 18 | 61 |
| 30 | **Yazd** | 1481 | 131 | 8.85 | 11.51 (9.70-13.32) | 6 | 4.58 | 71 | 60 | 18 | 24 | 4 | 6 | 20 | 1 | 4 | 15 |
| 31 | **Zanjan** | 913 | 183 | 20.04 | 17.78 (14.97-20.59) | 3 | 1.64 | 123 | 60 | 17 | 15 | 1 | 0 | 21 | 1 | 1 | 38 |
|  | **Total** | **81709** | **9,146** | **11.19** | **11.56 (11.21-11.91)** | **398** | **4.35** | **5,073** | **4,073** | **1,320** | **793** | **310** | **91** | **1,210** | **130** | **121** | **881** |
|  | * Per 100,000; CI: Confidence Interval, CFR: Case Fatality Rate * 100, CVD: Cardiovascular Disease, CRD: Chronic Respiratory Disease, CLD: Chronic Liver Disease, COPD: Chronic Obstructive Pulmonary Disease. | | | | | | | | | | | | | | | | |
