# Supplementary material for: Geospatial epidemiology of hospitalized patients with a positive influenza assay: A nationwide study in Iran, 2016–2018
Source: PLoS One. 2022 Dec 13;17(12):e0278900. doi: 10.1371/journal.pone.0278900 (PMC9747007; doi:10.1371/journal.pone.0278900)
Supplement: Attachment — Submitted filename: Dear Editor.docx [file pone.0278900.s003.docx]

**Dear Editor**

Thank you for inviting me to review the manuscript entitled “Geospatial Epidemiology of Hospitalized Patients with a Positive Influenza Assay: A Nationwide Study in Iran, 2016-2018”.

I read the manuscript and two important comments which are as follows:

1. In the method section, it has been written that multivariate logistic regression has been used for analysis. As you know, how variables are coded plays a very important role in interpreting the results of this regression. But in this methodology, how to encode any of the variables entered into the regression model has not been stated.

2. In the method section, it has been written the data of confirmed cases (*i.e.* positive PCR) has been used to perform calculations and draw a map of the spatial distribution of influenza. The first point is that in the executive field, performing PCR diagnostic test for influenza cases is not common and this test is performed only at the request of the patient’s treating specialist in certain circumstances (for example, the person is likely to be hospitalized in the ICU and to achieve a definitive diagnosis and to avoid infecting other critically ill patients admitted to the ICU on a case-test basis, PCR test is requested), which also this is not a routine measure of influenza diagnosis in most hospitals in the country, and the “clinical diagnosis” of a physician is the criterion for hospitalization. The second point is that a significant number of possible hospitalized cases of influenza are eventually coded under the heading of severe respiratory syndrome, respiratory disorder, or pneumonia according to “ICD-10” at the time of discharge, and their coding is not precisely and specifically done for influenza, and therefore a significant number of patients with definitive influenza that forms a part of data, is not reported in this article due to registration in another code (for example severe respiratory syndrome, respiratory disorder, pneumonia). The third point is that around 2018, for example, due to numerous “clinical diagnosis” reports of influenza and flu-like illness cases and the decision of the medical system to confirm the diagnosis and rapid response to the outbreak, some provincial medical universities (such as Gorgan, and East Azerbaijan provinces) were allowed to perform PCR tests to diagnose suspected hospitalizations. Therefore, the number of tests and, consequently, the number of positive cases identified due to these tests increased, but similar tests has not been done in some of the Iranian provinces or has not been given much attention which might indeed lead to unrepresentative data.

I believe that, all of the above-mentioned items cause that the estimates and maps obtained from this data do not reflect the real picture of the problem, and also is prone to significant underestimation. For non-professional readers who are not familiar with conventional clinical processes, may be lead to misrepresenting high-risk and low-risk areas.
