# Supplementary material for: Geospatial epidemiology of hospitalized patients with a positive influenza assay: A nationwide study in Iran, 2016–2018
Source: PLoS One. 2022 Dec 13;17(12):e0278900. doi: 10.1371/journal.pone.0278900 (PMC9747007; doi:10.1371/journal.pone.0278900)
Supplement: Attachment — Submitted filename: PONE-D-22-08786R1.docx [file pone.0278900.s005.docx]

Dear Editor;

Thank you for inviting me to review the manuscript number PONE-D-22-08786R1.

As already mentioned, my comments are methodological in nature; and the fundamental objection to the nature of the data used in this study. In other words, based on the usual implementation method in hospitals, the data of this article is a “non-representative sample”. This problem has not been noticed by the authors, so that neither the main findings, nor the short title, nor the abstract of the article mention this important issue. In addition, the authors clearly concluded that influenza hotspot clusters have been identified. Considering the fact that this study’s data is a non-representative sample, this claim is incorrect.

Considering all the above-mentioned, I suggest to the editor to **reject** the manuscript. But if the editor's opinion is not like this, at least the authors should be asked to add the number of PCR tests performed in each province during the study period. It can be used as bases for comparison and to understand why the claim of identifying influenza hotspot clusters is incorrect.
