# Supplementary material for: Geospatial epidemiology of hospitalized patients with a positive influenza assay: A nationwide study in Iran, 2016–2018
Source: PLoS One. 2022 Dec 13;17(12):e0278900. doi: 10.1371/journal.pone.0278900 (PMC9747007; doi:10.1371/journal.pone.0278900)
Supplement: Attachment — Submitted filename: Response to Reviewers.docx [file pone.0278900.s006.docx]

**Dear Dr. Hamid Sharifi;**

I appreciate you and the reviewers for examining our manuscript again. I would like to emphasize that we had mentioned in different places in our manuscript that our data is the representative of hospitalized PCR-confirmed cases of influenza, and we had not claimed that this is a total flu representative in the country. For instance, if one checks our manuscript title, our study subjects are those hospitalized patients with PCR-confirmed tests of Influenza. As a result, we have not claimed that our study is the whole picture of total Influenza or Influenza-Like Illness in the country. That would be another study with a different dataset which is the reviewer’s concern. We have responded to the reviewer’s comment as follows. Also, some revisions have been performed according to the comment.

Sincerely,

On behalf of all authors,

**Dr. Behzad Kiani**

École de Santé Publique de L’Université de Montréal (ESPUM), Québec, Montréal, Canada. Email: [kiani.behzad@gmail.com](mailto:kiani.behzad@gmail.com)

**Reviewer's comments:**

**Reviewer #3:**

**Reviewers’ comment**: As already mentioned, my comments are methodological in nature; and the fundamental objection to the nature of the data used in this study. In other words, based on the usual implementation method in hospitals, the data of this article is a “non-representative sample”. This problem has not been noticed by the authors, so that neither the main findings, nor the short title, nor the abstract of the article mention this important issue. In addition, the authors clearly concluded that influenza hotspot clusters have been identified. Considering the fact that this study’s data is a non-representative sample, this claim is incorrect. Considering all the above-mentioned, I suggest to the editor to **reject** the manuscript. But if the editor's opinion is not like this, at least the authors should be asked to add the number of PCR tests performed in each province during the study period. It can be used as bases for comparison and to understand why the claim of identifying influenza hotspot clusters is incorrect.

**Response:** Thanks for your comment and your time in examining our manuscript again. We respect your concern, but we have not claimed that our study is representative of total influenza in the country. As the study’s title shows, our study is spatial epidemiology of hospitalized patients with PCR-confirmed tests in Iran. So, when a reader reads our title, it is clear that our study is different from total influenza in terms of two limitations: first, all patients were hospitalized, the second, all of them were confirmed by PCR test. Also, we had mentioned in the Abstract > Introduction and Abstract > Methods that our study scope is related to hospitalized and PCR-confirmed cases. We think when somebody reads the title and abstract, it is absolutely clear what our study subjects are. However, according to your comment and to clarify our study scope better for the readers, we have revised our manuscript as follows:

1- The short title has been revised as follows:

*Geospatial epidemiology of lab-confirmed hospitalized influenza patients.*

2- Also, the Abstract > Conclusion has been revised as follows:

*We characterized the spatial and epidemiological heterogeneities of severe hospitalized influenza cases confirmed by PCR in Iran. Detecting influenza hotspot clusters could inform prioritization and geographic specificity of influenza prevention, testing, and mitigation resource management including vaccination planning in Iran.*

3- We have revised the limitation part as follows:

*We acknowledge that the results of this study are likely “non-representative” of the entire population.*

4- According to your request, we have uploaded the PCR data for each province via **S1 Appendix**.

We think these clarifications are enough because it is not concise to write hospitalized and PCR-confirmed influenza cases everywhere in the manuscript when we have mentioned it many times before.
